# Epidemiology of Candidemia: Three-Year Results from a Croatian Tertiary Care Hospital

**DOI:** 10.3390/jof7040267

**Published:** 2021-03-31

**Authors:** Ivana Mareković, Sanja Pleško, Violeta Rezo Vranješ, Zoran Herljević, Tomislav Kuliš, Marija Jandrlić

**Affiliations:** 1 Department of Clinical and Molecular Microbiology, University Hospital Centre Zagreb, School of Medicine University of Zagreb, 10000 Zagreb, Croatia; sanja.plesko@kbc-zagreb.hr (S.P.); violetarezovranjes@yahoo.com (V.R.V.); zoranhe@gmail.com (Z.H.); marija.jandrlic@gmail.com (M.J.); 2 Department of Urology, University Hospital Centre Zagreb, School of Medicine University of Zagreb, 10000 Zagreb, Croatia; tkulis@gmail.com

**Keywords:** candidemia, epidemiology, blood culture, central venous catheter, risk factors

## Abstract

Invasive candidosis is the most common invasive fungal infection in hospitalized patients and is associated with a high mortality rate. This is the first study from a Croatian tertiary care hospital describing epidemiology, risk factors and species distribution in patients with candidemia. A three-year retrospective observational study, from 2018 to 2020, was performed at the University Hospital Centre Zagreb, Zagreb, Croatia. A total of 160 patients with candidemia (*n* = 170 isolates) were enrolled. Candidemia incidence increased from 0.47 to 0.69 per 1000 admissions in 2018 and 2020, respectively. Ninety-five patients (58.38%) were in the intensive care unit. The main risk factors for candidemia were central venous catheter (CVC) (84.38%), previous surgical procedure (56.88%) and invasive mechanical ventilation (42.50%)*. Candida albicans* was identified in 43.53% of isolates, followed by *C. parapsilosis* (31.76%) and *C. glabrata* (12.36%), *C. krusei* (5.29%), *C. tropicalis* (2.35%) and *C. lusitaniae* (2.35%). The study discovered a shift to non-*albicans* *Candida* species, particularly *C. parapsilosis*, and made it possible to determine the main tasks we should focus on to prevent candidemia in the hospital, these being mainly infection control measures directed towards prevention of catheter-related bloodstream infections, specifically comprising hand hygiene and CVC bundles of care. The potential benefit of fluconazole prophylaxis in certain populations of surgical patients could also be considered.

## 1. Introduction

Invasive candidosis (IC) is the most common invasive fungal infection in hospitalized patients, especially in tertiary care hospitals. Despite advances in the medical care of critically ill patients, IC is still considered difficult to diagnose, causes prolonged hospitalization and a mortality rate ranging from 20% to 40% and poses a significant financial burden for hospital healthcare systems. Although IC refers to bloodstream infections and deep-seated infections, including intra-abdominal abscess, peritonitis or osteomyelitis, the majority of epidemiological surveys on IC are still based solely on documented bloodstream infections—candidemia. This may be because this is the most common form of IC but also due to the difficulties in diagnosing deep-seated infections without candidemia [1].

The majority of invasive infections are caused by five pathogens: *Candida albicans*, *Candida glabrata*, *Candida tropicalis*, *Candida parapsilosis* and *Candida krusei*. *C. albicans* continues to be the most prevalent *Candida* spp. in both adult and pediatric populations. However, over the last decades, species distribution has been changing, with a decrease in a proportion of *C. albicans* and an increase in *C. parapsilosis* and *C. glabrata* as a consequence of antifungal use and specific patients’ risk factors. This shift from *C. albicans* to non-*albicans Candida* spp. has been observed in epidemiological studies worldwide, where non-*albicans Candida* spp. represent >50% of the bloodstream isolates [2,3,4,5,6].

The global incidence of candidemia has increased in the last decade and is dependent upon geographical location and patient population. Recent global estimates have suggested that roughly 700,000 cases of invasive candidiasis occur annually. There are studies from many European countries analyzing the epidemiology of candidemia, showing differences between countries and underscoring the necessity of surveillance. These data are sometimes difficult to compare because there is extensive population-based surveillance performed in some countries while in others there are only small studies with patient days, patient discharges, hospital admissions or ICU admissions used as denominators. However, knowing the local incidence, risk patient populations and species distribution is a basis for adequate management of candidemia, including empirical antifungal treatment, prophylaxis and infection prevention and control measures [7].

Different well-described risk factors have been recognized to be associated with candidemia: patient in intensive care unit (ICU) with or without assisted ventilation, central venous catheter, total parenteral nutrition, diabetes, renal replacement therapy, multiple abdominal surgery, necrotizing pancreatitis, etc. [8,9]. Identification of risk factors is necessary because this is the key to successful prevention and empirical treatment of candidemia in patients with the above mentioned risk factors, especially those who have been treated previously with broad-spectrum antibiotics.

*Candida* spp. rank in the top three or four pathogens causing health-care-associated bloodstream infections. COVID-19 extensively influenced patient care in the year 2020. Many studies have been published describing the demographic and clinical characteristics, as well as outcomes, of COVID-19 patients, including those in ICUs. However, little is known about infectious complications, including bloodstream infections, caused by other pathogens and their influence on the outcome of COVID-19 patients, especially those in ICUs. The interaction between COVID-19 and incidence of candidemia is currently under investigation. It is obvious that every study about the epidemiology of candidemia that includes the year 2020 should have an observation regarding the impact of COVID-10 on the incidence of candidemia [10].

Data on the epidemiology of candidemia in Croatia, either from tertiary care hospitals or at the national level, are scarce at the moment. This is the first study from Croatia, specifically from its largest tertiary care hospital, describing incidence, risk factors and species distribution in patients with candidemia.

## 2. Materials and Methods

This three-year retrospective observational study was performed at the University Hospital Centre Zagreb, Zagreb, Croatia, a 1795-bed tertiary care hospital, from January 2018 to December 2020. The study was approved by the Ethics Committee of the University Hospital Centre Zagreb.

Candidemia was defined as at least one positive blood culture for *Candida* spp., from at least one peripheral or central line blood culture, in patients with signs or symptoms of infection. Collected demographic and clinical patient data included age, gender, underlying diseases and risk factors for candidemia.

Isolation of *Candida* spp. from blood was undertaken with one of two blood culture systems, BACT/ALERT^®^VIRTUO (BioMerieux, Marcy-l’Étoile, France) and BACTEC FX (Becton Dickinson, Franklin Lakes, NY, USA), using aerobe and anaerobe blood cultures with incubation in the instrument for up to five days. Bottles flagged positive were subjected to Gram staining. Direct identification from positive blood culture was done with peptide nucleic acid fluorescent in situ hybridization (PNA-FISH) (AdvanDx, Woburn, MA, USA) and identification of isolates grown after subculture on solid media was performed with matrix-assisted laser desorption/ionization time-of-flight mass spectrometry (MALDI-TOF MS) (Bruker Daltonik GmbH, Bremen, Germany) to the species level.

Susceptibility testing to antifungal agents was performed by reference method broth microdilution and interpreted according to Clinical and Laboratory Standards Institute (CLSI) guidelines [11]. Only *Candida* spp. isolates from the period 2019–2020 were tested with this reference method because the method was introduced in the routine praxis of the lab during 2018. Statistical analysis included descriptive frequency tables and calculation of incidence per 1000 admissions. To analyze risk factors associated with specific *Candida* species, a multivariate logistic regression model was applied. A backward stepwise logistic regression was used, and selected variables were analyzed in the model using a cut-off *p* value of 0.10. Results are reported with the odds ratio (OR), 95% confidence interval (CI) and *p* value. Statistical analyses were performed using MedCalc for Windows, version 19.7.2 (MedCalc Software, Ostend, Belgium). Patients with mixed candidemia were not included in logistic regression statistical analysis.

## 3. Results

During the three-year study period a total of 160 patients with candidemia (*n* = 170 isolates) were identified in our hospital and the overall candidemia incidence increased from 0.47 to 0.69 per 1000 admissions in 2018 and 2020, respectively (Table 1).

The demographic and clinical characteristics of patients are shown in Table 2. Out of 160 patients with candidemia, 54.38% were male and 45.62% were female. The median age was 62 (mean age 51.65 years, range 0–86 years). Infants younger than 1 year of age accounted for 6.25% (10/160) and patients older than 70 years for 23.75% (38/160) of patients. Ninety-five of 160 patients (58.38%) were at the ICU at the time when candidemia was detected.

The main risk factors for candidemia were the presence of a central venous catheter (CVC) (84.38%), being in the intensive care unit (59.38) and having had a previous surgical procedure (56.88%). Among the 91 patients who had undergone a surgical procedure, 24.38% had abdominal surgery. Repeated surgical procedure was necessary in 54 patients (33.75%), mainly after abdominal surgery (23/54, 42.59%).

During the three-year period, *C. albicans* was identified in 43.53% of isolates, followed by *C. parapsilosis* (31.76%), *C. glabrata* (12.36%), *C. krusei* (5.29%), *C. tropicalis* (2.35%) and *C. lusitaniae* (2.35%) (Table 3). Other species represented < 1% of isolates. Mixed infections due to combinations of two different *Candida* spp. occurred in nine patients (5.63%). The combinations were *C. albicans* and *C. glabrata* (*n* = 2); *C. albicans* and *C. tropicalis* (*n* = 1); *C. albicans* and *C. lusitaniae* (*n* = 1); *C. lusitaniae* and *C. parapsilosis* (*n* = 1); *C. albicans* and *C. parapsilosis* (*n* = 1); *C. fabianii, C. parapsilosis* and *C. tropicalis* (*n* = 1); *C. albicans* and *C. guillermondii* (*n* = 1); and *C. albicans* and *C. krusei* (*n* = 1).

**Figure 1:**
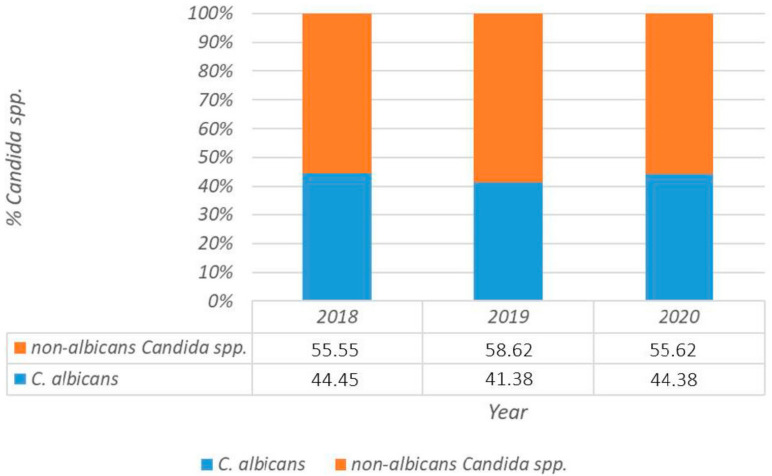
Proportion rate of *C. albicans* and non-*albicans Candida* species during the three-year study period.

Multivariate logistic regression analysis of risk factors for candidemia with the three most frequently isolated *Candida* spp. is shown in Table 4. The proportion rate of *C. albicans* and non-*albicans Candida* species during the three-year study period is shown in Figure 1.

The results of susceptibility testing of the three most frequently isolated *Candida* spp. to antifungal agents are shown in Table 5. Susceptibility dose-dependent or resistance to fluconazole was detected in 52 (51.49%) of the isolates, mainly in *C. parapsilosis* and *C. glabrata*. Resistance to the three echinocandins was found in six *C. albicans* isolates and one *C. parapsilosis* isolate. All *Candida* spp. isolates were susceptible to amphotericin B.

## 4. Discussion

This is the first study describing incidence, species distribution and risk factors in patients with candidemia in Croatia; it was performed retrospectively at the largest Croatian tertiary care hospital over a three-year period.

Studies from several European countries demonstrated an increase in the incidence of candidemia during the last decade, as was shown in our study for the last three-year period [3,4,5,6]. There is a reasonable explanation for this: the increasing number of complex surgical procedures, solid organ and haematopoietic stem cell transplantations, implantations of indwelling devices and patients treated with antineoplastic and immunosuppressive therapy, as well as the increasing number of elderly patients with underlying diseases. In a recent study analyzing the epidemiology of candidemia in Europe, the incidence rate for the total hospital-based setting was 0.83 per 1000 admissions per year, varying from 0.17 in Finland to 2.19 in Portugal [7]. Incidence rates observed in our study were within European frames. The functioning of healthcare systems, including hospitals, in 2020 was largely determined by the COVID-19 pandemic. According to published data, it is still not clear whether COVID-19 patients show a higher incidence of candidemia. Also, there is still no comparison of clinical presentation and outcomes between patients with and without COVID-19 available [12,13,14]. In our study in the year 2020, there were no COVID-19 patients among the patients with candidemia because the ward and intensive care unit for COVID-19 patients did not exist in the hospital until the end of 2020 and during our study period COVID-19 patients were transferred to other COVID-19-dedicated hospitals.

In various surveys conducted in Europe, about 40–50% of patients were at the ICU at the time of candidemia. Many studies that have discussed patients hospitalized in ICUs and determined risk factors for candidemia [15,16,17,18,19,20]. More than half of patients with candidemia (59.38%) in our study were ICU patients. Having a central venous catheter or previous surgical procedure were the most frequently present clinical characteristics, found in 135 (84.38%) and 91 (56.88%) patients with candidemia, respectively.

The significance of previous surgical procedures as a risk factor for candidemia, especially abdominal surgery, has been demonstrated in many previous publications [21,22,23,24]. Among 160 patients with candidemia in our study, 91 (56.88%) had undergone a previous surgical procedure, predominantly an abdominal type of surgery. According to current ESCMID guidelines, fluconazole prophylaxis is recommended in patients who have recently undergone abdominal surgery and have recurrent gastrointestinal perforations or anastomotic leakages. In our hospital setting, fluconazole prophylaxis is not routinely administered in this patient population [25].

Although *C. albicans* was the most common species isolated in our patients (43.53%) throughout the three-year period, there was also a high incidence of the second-ranked *C. parapsilosis*, accounting for a total of 31.76% of all isolates, followed by *C. glabrata* (12.35%). The proportion of *C. albicans* in our study dropped to less than 50% of all *Candida* spp. isolates, showing the shift to non-*albicans Candida* species. This finding has important clinical implications, as *C. parapsilosis* and *C. glabrata* show decreased susceptibility to azoles and echinocandins, respectively. Epidemiology studies from European countries show that this shift is not yet a universal characteristic as there are still countries in which *C. albicans* is the predominant species [26,27]. Our results show a *Candida* spp. distribution typical for southern Europe, with *C. parapsilosis* ranked second and *C. glabrata* third in frequency. A high percentage of *C. parapsilosis*, similar to our study, can also be found in Latin America and Africa. In studies from Italy and Spain, the incidence of this species ranged from 14.8 to 46.8% [15,28,29]. The explanation for this distribution is unknown, although it may be a consequence of climate influence, antifungal policy or central venous catheter care procedures [30]. In contrast, in northern Europe, the USA and Australia, *C. albicans* has been gradually replaced by *C. glabrata*, which ranks second in these geographical areas [31,32].

In our study, diabetes mellitus and invasive mechanical ventilation were independent risk factors for *C. albicans* candidemia. Several mechanisms provide the basis for the predisposition of diabetic patients for candidemia: yeast adhesion to epithelial cell surfaces, higher salivary glucose levels, reduced salivary flow, microvascular degeneration and impaired candidacidal activity of neutrophils. Some authors have also suggested that there is also higher *C. albicans* colonization of the oral cavity and intestines in these patients [33]. In patients on long-term mechanical ventilation, there are many coexisting factors (broad-spectrum antibiotics, intravenous steroids, etc.), making these patients prone to fungal colonization or invasive fungal disease. However, the routine and universal administration of antifungal prophylaxis in critically ill patients is not recommended [34,35].

Younger age and CVC were independent risk factors for *C. parapsilosis* candidemia. It is well-known that *C. parapsilosis* is a fungal agent that mostly affects patients with CVC hospitalized in ICUs, as well as the pediatric population, especially neonates. This species can be isolated from patients’ skin, the hands of healthcare workers and the hospital environment and is associated with colonization of catheters. Furthermore, *C. parapsilosis* genotypes recovered from hands were also found in blood samples, thus suggesting that the infections caused by *C. parapsilosis* might be, in part, attributable to horizontal transmission of this species by hospital staff [36,37]. In previous studies, prior antibiotic therapy parenteral nutrition, prior surgery, prior immunosuppressive therapy, malignancy, caspofungin treatment and transplant receipt have all been identified as risk factors for *C. parapsilosis* candidemia, showing that the rise of *C. parapsilosis* may be considered a consequence of advances in healthcare [38].

Older age and total parenteral nutrition (TPN) were independent risk factors for *C. glabrata* candidemia. *C. glabrata* has already been found to be more frequent in the elderly in previous studies, and there is not a clear explanation for this finding [30,39]. Regarding TPN, the data in the literature are so far inconclusive—there are studies showing the association of TPN with both *C. albicans* and non-albicans *Candida* species [40,41].

The most prominent finding regarding antifungal susceptibility was the high rate of fluconazole resistance of 83.33% (30/36) in *C. parapsilosis*. The emergence of fluconazole resistance in *C. parapsilosis* has been increasingly reported recently. Furthermore, according to previous studies, patients with candidemia due to resistant isolates had poorer outcomes in comparison to those with susceptible ones [42].

This is the first study from a Croatian tertiary care hospital describing the epidemiology of candidemia. As knowledge of candidemia epidemiology in Europe is based on the individual engagements of researchers in the field of mycology and microbiology, our study presents a significant contribution, as data from Croatia were missing so far on the candidemia map of Europe. The main contributions of this study are the discovered shift to non-*albicans* species, particularly *C. parapsilosis*, and the high rate of fluconazole resistance in *C. parapsilosis*. Although the epidemiology of candidemia is specific to each hospital/center, and even to each ward, this study may be an indicator of what we can expect in future studies published from other Croatian hospitals. Our results also make it possible to determine the main tasks we should focus on in order to prevent candidemia in the hospital. Specific tasks include further improvements in the implementation of infection control measures directed towards prevention of catheter-related bloodstream infections, specifically comprising hand hygiene and CVC bundles of care. Physicians should also be aware of the risk of candidemia in patients on long-term mechanical ventilation. The potential benefit of fluconazole prophylaxis in certain populations of surgical patients can also be considered. Further surveillance of epidemiological characteristics and candidemia rates in our hospital as well as at the national level is needed.

## Figures and Tables

**Table 1:** Number of patients with candidemia, *Candida* spp. isolates, hospital admissions and incidence rates in three-year study period (2018–2020)**.**

	2018	2019	2020
Number of patients with candidemia	50	58	52
Number of *Candida* spp. isolates	54	58	58
Hospital admissions	106,186	88,003	75,023
Incidence per 1000 admissions	0.47	0.66	0.69

**Table 2:** Demographic data and clinical characteristics of 160 patients with candidemia.

	Median:Mean (Range) or n (%)
Age (years)	62:51.65 (0–86)
Gender (male)	87 (54.38)
Clinical characteristic ^a^	
Central venous catheter	135 (84.38)
Intensive care unit	95 (59.38)
Previous surgical procedure	91 (56.88)
Type of surgical procedure	
Abdominal	39 (24.38)
Cardiac	13 (8.13)
Thoracic	14 (8.75)
Vascular	11 (6.88)
Neurosurgery	7 (4.38
Orthopedic	2 (1.25)
Multiple trauma	0
Solid organ transplant	1 (0.63)
Other	6 (3.75)
Repeated surgery	54 (33.75)
Invasive mechanical ventilation	68 (42.50)
Total parenteral nutrition	56 (35.00)
Solid tumor	40 (25.00)
Hematological malignancy	23 (14.38)
Dialysis at presentation	23 (14.38)
Diabetes mellitus	22 (13.75)
Pancreatitis	4 (2.50)
Burns	0
Human immunodeficiency virus	0

^a^ some patients had more than one clinical characteristic.

**Table 3:** Species distribution of *Candida* isolates in the three-year study period (2018–2020).

	2018	2019	2020	Total
*C. albicans*	24 (44.45%)	24 (41.38%)	26 (44.83%)	74 (43.53%)
*C. parapsilosis*	19 (35.19%)	21 (36.20%)	14 (24.15%)	54 (31.76%)
*C. glabrata*	7 (12.96%)	7 (12.07%)	7 (12.07%)	21 (12.36%)
*C. krusei*	1 (1.85%)	4 (6.90%)	4 (6.90%)	9 (5.29%)
*C. tropicalis*	1 (1.85%)	0	3 (5.17%)	4 (2.35%)
*C. lusitaniae*	2 (3.70%)	2 (3.45%)	0	4 (2.35%)
*C. dubliniensis*	0	0	1 (1.72%)	1 (0.59%)
*C. fabianii*	0	0	1 (1.72%)	1 (0.59%)
*C. guilliermondii*	0	0	1 (1.72%)	1 (0.59%)
*C. kefyr*	0	0	1 (1.72%)	1 (0.59%)
Total	54	58	58	170

**Table 4:** Independent risk factors associated with the three most frequently isolated *Candida* spp. (*n* = 151).

	*C. albicans* (*n* = 67)			*C. parapsilosis* (*n* = 51)			*C. glabrata* (*n* = 19)		
	OR	95% CI	*p*	OR	95% CI	*p*	OR	95% CI	*p*
Age				0.98	0.97–1.0	0.011	1.05	1.01–1.08	0.011
Diabetes mellitus (21)	4.21	1.48–12.3	0.007						
Total parenteral nutrition (53)	0.38	0.15–0.97	0.042				5.09	1.24–20.84	0.024
Invasive mechanical ventilation (64)	2.17	0.88–5.40	0.094				0.20	0.05–0.86	0.030
Central venous catheter (126)	0.40	0.16–1.05	0.063	6.07	1.60–23.01	0.008			

OR: odds ratio, CI: confidence interval; the cut-off *p* value for variables selected in the backward stepwise logistic regression was 0.10.; patients with mixed infections were not included in the analysis.

**Table 5:** Susceptibility to antifungal agents of the three most frequently isolated *Candida* spp. in the period 2019–2020.

			Number (%) of Isolates	
Species (Number of Isolates)	Antifungal Agent	Susceptible	Susceptible Dose-Dependent	Resistant
*C. albicans* (51)	Fluconazole	49 (96.08)	0	2 (3.92)
	Anidulafungin	49 (96.08)	1 (1.96)	1 (1.96)
	Caspofungin	48 (94.12)	0	3 (5.88)
	Micafungin	49 (96.08)	0	2 (3.92)
	Amphotericin B	51 (100.0)	0	0
*C. parapsilosis* (36)	Fluconazole	6 (16.67)	6 (16.67)	30 (83.33)
	Anidulafungin	30 (83.33)	5 (13.89)	1 (2.78)
	Caspofungin	36 (100.0)	0	0
	Micafungin	34 (94.4)	2 (5.56)	0
	Amphotericin B	36 (100.0)	0	0
*C. glabrata* (14)	Fluconazole	0	10 (71.43)	4 (28.57)
	Anidulafungin	14 (100.0)	0	0
	Caspofungin	13 (92.86)	1 (7.14)	0
	Micafungin	14 (100.0)	0	0
	Amphotericin B	14 (100.0)	0	0
